# The H-factor as a novel quality metric for homology modeling

**DOI:** 10.1186/2043-9113-2-18

**Published:** 2012-11-02

**Authors:** Eric di Luccio, Patrice Koehl

**Affiliations:** 1 Computer Science Department, University of California Davis, 451 East Health Sciences Drive, Room 4337, Genome Center, GBSF, Davis, CA, 95616, USA; 2 School of Applied Biosciences, Kyungpook National University, Agriculture and life science building #3, room 309, 1370 Sangyeok-dong, Buk-gu, Daegu, 702-701, Republic of Korea

## Abstract

**Background:**

Drug discovery typically starts with the identification of a potential target that is then tested and validated either through high-throughput screening against a library of drug compounds or by rational drug design. When the putative target is a protein, the latter approach requires the knowledge of its structure. Finding the structure of a protein is however a difficult task. Significant progress has come from high-resolution techniques such as X-ray crystallography and NMR; there are many proteins however whose structure have not yet been solved. Computational techniques for structure prediction are viable alternatives to experimental techniques for these cases. However, the proper validation of the structural models they generate remains an issue.

**Findings:**

In this report, we focus on homology modeling techniques and introduce the H-factor, a new indicator for assessing the quality of protein structure models generated with these techniques. The H-factor is meant to mimic the R-factor used in X-ray crystallography. The method for computing the H-factor is fully described with a demonstration of its effectiveness on a test set of target proteins.

**Conclusions:**

We have developed a web service for computing the H-factor for models of a protein structure. This service is freely accessible at http://koehllab.genomecenter.ucdavis.edu/toolkit/h-factor.

## Background

Structure-based drug design relies on the concept of “druggability” which is used to describe proteins that possess structures that favour interactions with a drug-like chemical compound. Many “druggable” proteins (as identified from their structures) however are not drug targets, as binding does not always guarantee therapeutic activity. To predict if a “druggable” protein can be a target requires knowledge of its structure, of its dynamics, and of all regulatory mechanisms that control its expression and function. Ultimately, the knowledge of a high-resolution structure for the protein is essential, an information that is not yet available for many proteins. X-ray crystallography and NMR remain the experimental techniques of choice to acquire this knowledge; there are many proteins however that are difficult to crystallize or to purify to the level requested by these techniques. For these proteins, computational structure prediction methods are considered a viable alternative.

*In silico* protein structure prediction techniques fall into two categories: the *ab initio* folding methods and homology modelling. Both techniques routinely yield astounding results but do require caution, as models need to be thoroughly validated prior to use. Because the quality of models tends to be highly dependent of the available experimental data, tools, protocols and skills of the operator, validation metrics are needed to safeguard their usage. In this study, we focus on homology modelling, following our previous study where we reviewed the common practices in homology modelling of proteins and provided a set of guidelines for building better models [1]. Homology modelling predicts the structure of a protein exploiting the knowledge of a homologous protein whose structure is known. Its general strategy for predicting the structure of a target protein proceeds through a canonical seven-steps procedure: (1) Identify the template proteins that share structural similarity to the target; (2) Align the target sequence with the templates sequences; (3) Build a single framework of spatially aligned template structures and assimilate the target protein backbone with this framework; (4) Build the missing backbone elements (loops) not represented in the template framework; (5) Build the target side chains; (6) Refine the model in order to minimize unrealistic contacts and strains; and (7) Evaluate the final refined model for physical tenability.

Several homology modelling programs such as MODELLER [2], SegMod/ENCAD [3], Swiss-model [4], 3D-Jigsaw [5], BUILDER [6] and Nest [7] are commonly used to generate models in addition to online portals such as the Protein Structure Initiative (PSI) model portal or Swiss-Model Repository [8]. One of the drawbacks of using automated model-building programs is the lack of human interaction to detect possible anomalies that may render the model inaccurate or wrong. In our previous study, we made the demonstration of the dramatic effect of a single error in the sequence alignment by a single shift of one amino acid leading to a distortion of 3.8 Å in the backbone models [1]. Such distortion is sufficient to introduce significant bias in a binding site of an enzyme for instance, rendering any virtual ligand screening hopeless.

**Figure 1:**
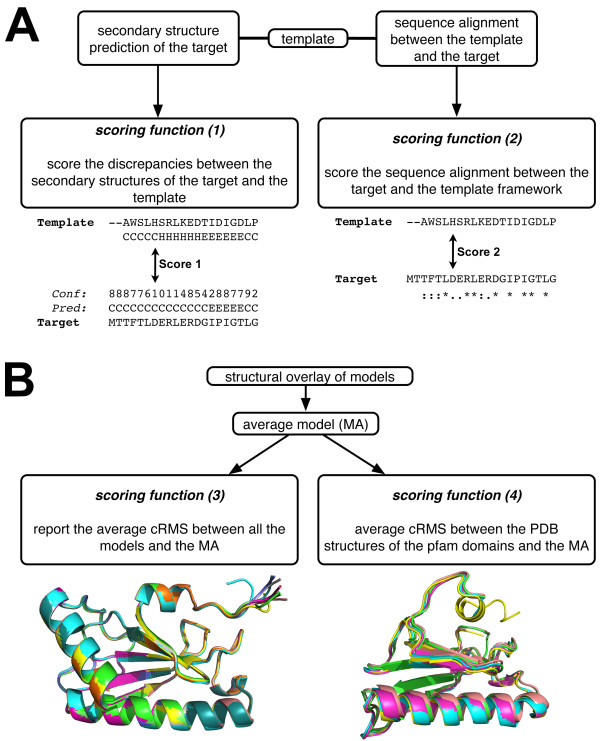
**Flowchart for computing the H-factor. A**. The scoring functions (**1**) and (**2**) are sequence-based. The score (**1**) compares the secondary structure prediction for the target sequence with the actual secondary structure assignment of the template protein. As an example, an extract of a sequence alignment between a template and a target is represented. The secondary structures of the template are indicated underneath the sequence of the template (C: coil, H: helix). Above the sequence of the target is indicated the secondary structure prediction for the target along with its confidence factor returned by PSIPRED. Score (**2**) evaluates the sequence similarity between the target and template sequence. **B**. The scoring functions (**3**) and (**4**) evaluate the structural models: score (**3**) quantifies the structural diversity among the models, while score (**4**) identifies the pfam domains in the target protein, collects the structures of these domains from the models to be tested, and compares these structures with those observed for the same domains in the PDB.

Difficulties in evaluating the correctness of models and specifically, the lack of cross validation indicators such as the R-factor and R-free in X-ray crystallography, hamper the proper use of homology modelling [9]. The quality assessment of models has been the focus of numerous studies and various algorithms exist with scoring functions based on statistical potentials [10], local side-chain and backbone interactions [11], residue environments [12], packing estimates [13], solvation energy [14], hydrogen bonding, and geometric properties [15]. In addition, the proper stereochemistry of models can be assessed by commonly used programs such as Procheck or WhatIf [16,17]. Despite all these methods, the homology modelling community still lacks a simple and easy to use indicator which gives an unambiguous feedback on how the final model, or family of models, reflects the data that were used in the modelling process, similar to the couple R-factor/R-free for X-ray crystallography. For that purpose, we introduced a new indicator for assessing the quality of homology models, namely the H-factor, that mimics the R-factor in X-ray crystallography [1] (Figure 1). This short report is a follow-up of this original work. In it, we provide a new description of the strategy used to compute the H-factor and describe new test cases that illustrate its effectiveness. 

### Computational strategy

The H-factor combines information of four scoring functions that evaluates (**1**) the template structure(s) (based on the corresponding PDB files); (**2**) the sequence alignment between the template(s) and the target sequences; (**3**) the structural heterogeneity of the models built; and (**4**) the structural neighborhood within protein families (Figure 1).

#### Score (1): Secondary structures analysis

The scoring function (**1**) analyses the discrepancies between the secondary structure prediction for the target obtained with the program psipred [18,19] and the actual secondary structures of the template framework computed with the program stride [20]. The corresponding score takes into account the confidence factors reported by psipred:

(1) $$\text{score1=}a\sum_{i=1}^{N}\frac{f\left(i\right)}{N}+b\;;\;f\left(i\right)=\{\begin{array}{l} 0\;\text{for}\;p=s\\ \frac{c\left(i\right)+1}{10}\;\text{for p}\neq s\\ 1\;\text{for sequence alignment gaps} \end{array}$$

The sum is computed over all positions in the sequence alignment between the target and template, *N* is the length of the sequence alignment, *p* is the secondary structure prediction of the target at position *i*, *c*(*i*) is the confidence factor reported by psipred for the secondary structure prediction at position *i* and *s* is the secondary structure type observed at position *i* in the template structure reported by stride. The offset coefficients *a* and *b* are set to 1.3 and 0.9, respectively, to ensure that score (**1**) has values between 0 and 10.

#### Score (2): Evaluation of the sequence alignment

The function (**2**) scores the identity between the sequence of the target and the sequence of the template framework where *N* is the length of the sequence alignment.

(2) $$\text{score2=}10\left(1-\frac{\sum_{i=1}^{N}g\left(i\right)}{N}\right)\;;\;g\left(i\right)=\{\begin{array}{l} 0\;\text{for non identity}\\ 1\;\text{for identity} \end{array}$$

#### Score (3): Measurement of the heterogeneity of generated models

The score (**3**) is designed to measure the heterogeneity of a set of generated models, which may be induced by either an improper or remote template. It is computed using all the models M_i_, as well as the corresponding average model, MA, whose atomic coordinates are the averages of the corresponding coordinates in the models. The function (**3**) then reports the average cRMS between each model and the average model, where the cRMS is computed over the Cα atoms only. The average cRMS is then transformed linearly such that the final score is between 0 and 10:

(3) $$\text{score3=}a\;\left(\frac{\sum_{\text{i=}1}^{\text{n}}{\text{cRMS(M}}_{\text{i}}\text{,MA})}{n}\right)+b$$

*n* is the number of models. The offset coefficients a and b are chosen such that average RMS values of 0.1 and 7 Å correspond to scores of 1 and 10, respectively; the corresponding values are *a* = 1.3 and *b* = 0.87.

#### Score (4): Assessment of the structural integrity of functional domains

Score (**4**) is designed to specifically evaluate the quality of all functional domains in the model with respect to available experimental structures deposited in the protein data bank (PDB). First, the various domains in the sequence are identified using HMMER [21,22] and the pfam profiles database as a reference [23], The average model MA is then broken down into fragments corresponding to these domains. Each fragment is compared to the structures of the same domain found in proteins whose structures have been deposited in the PDB. A maximum of 5 fragments is considered (the top 5 HMMER search results). To minimize the numbers of false positive we set an E-value cut-off of 1.0e-10 for hmmsearch. The score (**4**) is then the average cRMS distance between the fragments and their counterparts in the PDB:

(4) $$\text{score4=}a\;\left(\frac{\sum_{d=1}^{m}\sum_{\text{i=}1}^{\text{n}}{\text{cRMS (MA}}_{\text{d}}{\text{,D}}_{\text{d,i}})}{mn}\right)+b$$

*m* is the number of functional domains identified in the target sequence, MA_*d*_ is the structural fragment extracted from the average structure MA corresponding to the domain *d*, *n* is the number of domains homologous to domain *d* found in PDB structures, and D_d,i_ is the i-th possible structure of the domain homologous to *d*. The offset coefficients a and b have been chosen such that average RMS values of 0.1 and 7 Å correspond to scores of 1 and 10, respectively; the corresponding values are *a* = 1.3 and *b* = 0.87. This enforces that score (**4**) is between 0 and 10. Note that if this procedure does not find an equivalent domain with a known structure for a fragment, the fragment is ignored; if no domains are found for all fragments, score (**4**) is ignored.

The H-factor score is simply the average of scores (**1**, **2**, **3**, **4**). The H-factor computation is accessible online at http://koehllab.genomecenter.ucdavis.edu/toolkit/h-factor.

## Results and discussion

**Figure 2:**
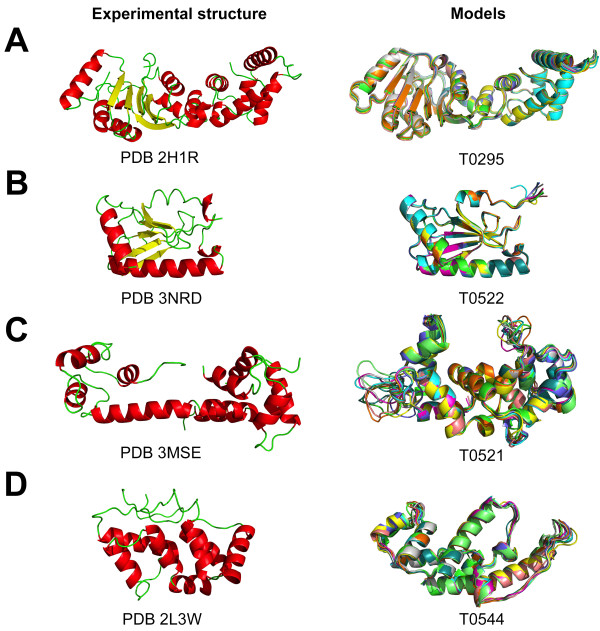
**Comparison of experimental structures with their respective homology models.***Left side panel*: experimental X-ray structures of selected CASP7 (**A**) and CASP9 (**B**, **C**, **D**) targets. *Right side panel*: structural overlay of ten models for each selected CASP target build with the closest available template in the protein data bank. **A** &**B** depicts “easy” modelling cases, while **C** &**D** are dramatically more challenging.

Target proteins of the CASP experiments (http://predictioncenter.org) are ideal test cases to benchmark the H-factor. In this short report, we have chosen targets from both the CASP7 and CASP9 experiments to demonstrate the usefulness of the H-factor as well as to highlight its sensitivity in identifying anomalies within homology models. The first CASP7 target chosen (T0295) is considered “easy”, while the second and third targets (T0522 and T0521) are more difficult cases for homology modelling (Table 1 and Figure 2). The fourth target (T0544) is the most difficult test case we have considered due to the lack of related templates available in the protein data bank.

**Table 1:** Comparison between the H-factor, cRMS, DOPE and QMEAN scores

**CASP targets**	**Scoring function (a)**				**H-factor (%)**	**cRMS (Å) (b)**	**% ID (c)**	**DOPE (d)**	**QMEANnorm (e)**
	**(1)**	**(2)**	**(3)**	**(4)**					
T0295	1.0	1.9	1.3	3.8	19	1.67	46	-33940	0.735
T0295* (f)	1.0	1.9	1.9	3.8	21	1.71	46	-24317	0.196
T0522	2.0	6.2	1.5	5.0	37	2.19	38	-14185	0.771
T0522* (f)	2.0	6.9	2.0	5.2	40	2.71	38	-12687	0.681
T0521	4.2	7.5	3.4	5.4	51	4.09	24	-18484	0.695
T0521^#^ (g)	6.1	7.6	3.6	6.8	60	4.24	23	-15968	0.508
T0544	7.0	8.3	4.0	8.3	69	6.11	17	-10652	0.192

(a) The four scoring functions included in the H-factor measure the quality of the secondary structure prediction (score (**1**)), the diversity of the sequence alignment (score (**2**)), the structural diversity of the models generated (score (**3**)), and the similarity of the predicted structures for the functional domains in the target, compared to the structures of the same domains found in the PDB (score (**4**)).

(b) Average cRMS (over Cα) between the 10 different models generated and the actual experimental structure for the target.

(c) Sequence identity between the target sequence and the template sequence.

(d) DOPE scores from MODELLER [24].

(e) QMEAN normalized score [25].

(f) T0295^*^ and T0522* are experiments in which the sequence alignment between the target and its framework has been deliberately modified with a shift of a single amino acid in the sequence alignment (see text for details).

(g) T0521^#^ is an experiment in which a less suitable template has been deliberately chosen compared to T0521 (see text for details).

For each CASP target considered, the best template structures were identified using fold recognition techniques. The top template for each target was then selected according to the CASP 7 & 9 analyses of every possible template for each target. We aligned the sequence of the target with its template(s) using Clustal W [26], with the Gonnet250 matrix to define the substitution score and default settings for gap penalties. This corresponds to a simple pairwise sequence alignment. We used MODELLER 9v5 [2], with the “automodel” settings to generate 20 models for each of the four targets. As the H-factor focuses only on the Cα-backbone, we did not attempt to improve the prediction of the sidechains.

The CASP7 target T0295 is an “easy” modeling case. It has a very close homologue (sequence identity 46%) whose structure has been solved at high resolution (PDB code: 1ZQ9, 1.9 Å resolution) and the template sequence covers its whole sequence. Using default settings, the non-specialist would have no difficulties in generating “good” models of T0295. The average cRMS (based on Cα only) between the T0295 models and the actual experimental X-ray structure for target T0295 (available in the PDB as 2H1R; resolution 1.89 Å) is 1.67 Å, indicating models of good quality. The corresponding H-factor for these 20 models is 19%, i.e. a very good score (by definition, H-factors vary between 0% and 100%, with 0% being good and 100% being bad). The good qualities of the T0295 models are highlighted by each scoring function included in the H-factor (Table 1). The secondary structure prediction for T0295 matches well with the actual secondary structure of its framework (1ZQ9), yielding a value of 1 for score (**1**). The sequence alignment between T0295 and 1ZQ9 is deemed good, with a value of 1.9 for score (**2**). The 20 models generated showed little structural dispersion with a corresponding value for score (**3**) of 1.3. Score (**4**) compared the structure of this domain in the models generated for T0295 and the structures of the same domain found in all five proteins listed above. It detected fluctuations between these structures, leading to a score of 3.8. The score (**4**) is relatively higher than the other scores; it does remain however within a range that indicates a good match. Note that the overall H-factor value is 19%. In comparison, a R-factor of 20% is typically observed for fully refined X-ray structures around 2 Å of resolution, i.e. for a good X-ray structure.

To further benchmark the sensitivity of the H-factor, we deliberately introduced a single shift in the alignment between the sequence of T0295 and the sequence of its template 1ZQ9 (see our previous report [1]). The corresponding models generated by MODELLER show structural diversity in the loop region near the shift. Score (**3**) captures this structural diversity within a set of models. It leads to the H-factor being raised from 19% to 21% (see Table 1). However, score (**2**) could not detect a single position shift in the alignment. The H-factor is therefore capable of detecting backbone deviation due to modeling errors, the same way the R-factor does.

The CASP9 target T0522 is a more difficult modeling case. The template sequence has low similarity with the target sequence and this is highlighted by the scoring function (**2**), which returns a value of 6.2 (out of 10) (Table 1). Note that the score (**2**) is not a direct measurement of the quality of the sequence alignment. It is designed to quantify the differences between the two sets of sequences: if these differences are small, the model is expected to be good, while if the differences are large the models should be considered with caution. The overall H-factor for the models generated for T0522 is 37%. This mid-range value indicates that caution should be used when interpreting or using these models. Indeed, the average cRMS between these models and the actual structure of T0522 (available in the PDB in the file 3NRD) is 2.19 Å, i.e. reflecting a medium-resolution agreement (Figure 2).

The main difficulty encountered in modeling T0522 was the proper sequence alignment between the template and the target as the sequence of T0522 is somewhat remote from its chosen template (PDB 3OHE). In modeling cases similar to T0522, the non-specialist may have difficulties in either generating a proper sequence alignment or assessing critically the end-result. To further demonstrate the effects of errors induced by improper sequence alignments, we deliberately introduced a one amino-acid shift in the alignment resulting in the H-factor rising from 37 to 40%. The score (**2**) jumped from 6.2 to 6.9 along with the score (**3**) from 1.5 to 2.0 highlighting an increase in model heterogeneity. The overall increase in errors is captured by an H-factor of 40% (raising from 37%), which indicates that the models contain errors and may deviate notably from the experimental structure.

The CASP9 target T0521 is a significantly more difficult modeling case compared to T0522. The main difficulty lies in identifying the “best” template. Only two remotely homologous structures were detected, with PDB codes 3PM8 and 2AAO and sequence identities of 24% and 23%, respectively. The non-specialist may face a problem choosing the template in addition to generating the best possible sequence alignment. To illustrate the structural errors induced by choosing a remote template, we built two sets of models for T0521 with either 3PM8 or 2AAO as template and assessed the errors with the H-factor (Table 1). In both cases, the sets of T0521 models contain significant errors in the secondary structure matches between the template and the target (score (**1**)) and the quality of the sequence alignment, score (**2**). In addition, score (**4**) indicates that the fold of the functional domains deviates from the available experimental structures deposited in the PDB. Overall the H-factors of these two sets of T0521 models are 51% and 60%, a good indication that these are incorrect models that do not represent the native structure of T0521 (Table 1). Indeed, the corresponding average cRMS values between the models generated with the templates 3PM8 and 3AAO and the native structure for T0521 are 4.09 and 4.24 Å, respectively highlighting gross differences with the native structure (Figure 2).

The CASP9 target T0544 is the most difficult test case we have considered due to the lack of related template available in the PDB. A database search over all sequences of proteins whose structure is known identifies a unique template, 3BRJ, with a low sequence identity of 17% (Table 1). In this specific case, all four scores reported high values (7.0, 8.3, 4.0 and 8.3 for scores (**1**), (**2**), (**3**) and (**4**), respectively). The overall H-factor is 69%, a value that should raise serious concerns about the quality of these models. The average cRMS between these models and the actual structure for T0544 (PDB 2L3W) is 6.1 Å, indicating that the models are very poor approximations of the native structure (Figure 2).

From the four test cases T0295, T0521, T0522 and T0544, we conclude that the H-factor correlates well with the quality of the models tested. The H-factor computes the quality of models for protein structures based on sequence information (score (**1**) and score (**2**)), as well as based on structure information (score (**3**) and score (**4**)). While the former is specific to homology modeling, the latter can be used to assess the quality of any sets of models. Although the H-factor and the R-factor are mathematically unrelated, they have the same purpose: to assess the quality of structures, either experimental or computed from modeling experiments, where quality refers to reflecting correctly the input data used to generate these structures. The H-factor mimics the R-factor as it provides a quality-index to follow in the process of building a model, the same way crystallographers monitor the R-factor/R-free indexes during structures refinements. In our previous study, we compared the H-factor results with the experimental R-factor and R-free on a randomly chosen subset of the PDB containing 445 structures with 6 or more identical chains solved by X-ray crystallography [1]. The H-factor and R-factor are not linear correlated but it remains that “good” R-factors (below 30%) correspond to “good” H-factor values (below 45%) [1]. The H-factor checks the diversity of the set of models generated for a structure, as well as their similarities with the structures of domains that share the same function, as defined by pfam. High H-factor values may be caused by structures with disordered loops or remote structural neighbors in the PDB. It remains that, the H-factor recognizes experimentally determined structures as being valid [1].

The statistical potential Discrete Optimized Protein Energy (DOPE) measure model quality and has been introduced in MODELLER v8 [24]. DOPE is a statistical potential with an improved reference state that accounts for the compact shape of native protein structures. The DOPE score is designed such that large, negative scores are usually indicators of good models. In their original study, Shi and Sali [24] found that the accuracy of DOPE to asses a homology model improves as the accuracy of the models improve. We observe a similar behaviour for targets T0295 and T0295* (Table 1). These two targets correspond to the same protein and it is therefore possible to compare the DOPE scores of their models. The model generates for T0295*, based on an incorrect alignment, has a much lower DOPE score (-24317) that the model generated with the correct alignment (T0295; -33940). Note that we cannot compare DOPE scores for proteins of different sizes, as these scores are not normalized. DOPE scores are therefore relative, and designed to pick a “good” model among poorer models. DOPE scores do not assess directly the quality of the model that is picked, i.e. if it is likely to be similar to the actual structure. The H-factor is a better indicator in that respect.

QMEAN, which stands for Qualitative Model Energy ANalysis, is a composite scoring function for homology models that describes the major geometrical aspects of protein structures as well as the agreement between the predicted and calculated secondary structure and solvent accessibility, respectively [25]. As such, it includes a term similar to the score (**1**) of the H-factor, as well as terms that assess different properties such as residue accessibility. The score QMEANnorm is a normalised version of the QMEAN score in which all terms are divided by the number of interactions/residue in order to avoid a size-bias of the score [25]. QMEANnorm scores vary between 0 and 1, with larger scores expected to correspond to better models. Unlike the DOPE score, both the H-factor and QMEANnorm scores allow for the comparison of proteins of different sizes. The QMEANscore is as effective as PROSA or DOPE for detecting errors in a model that result from errors in the sequence alignment between the template and target protein: T0295* has a QMEANnorm score of 0.196 while the score for T0295 is 0.735. Interestingly, T0522 (0.771) has a more favorable QMEANnorm score than the significantly accurate model generated for T0295 (0.735) (Table 1). We have observed however that the QMEANnorm score is prone to fail: some of the erroneous models generated for the CASP target T0521 and T0522 have QMEANscores of 0.771 and 0.695, respectively, meaning they are evaluated to be almost as correct as the positive control T0295 (0.735). Unlike QMEAN, the H-factor did detect that these models were to be considered with caution. Because it analyzes a set of models, we believe that the H-factor score is more robust as an absolute measure of the quality of a model. It lacks however the ability to discriminate among a set of models generated for the same target.

These results emphasize the essential differences in the nature of the ProSA, DOPE, QMEANnorm and H-factor scores. ProSA, DOPE and QMEAN check the quality of a model, independently of the context in which it was generated. The H-factor on the other hand checks the quality of a set of models with respect to a context that includes for example the sequence alignment assessed by the score (**2**). The modeler however should use these differences to extend his/her assessment of the model his/she generates. We believe that ProSA, DOPE, QMEAN and H-Factor analyses are needed to provide a better overview of the quality of models derived by homology modeling.

### Model verification and validation in structure-based drug-design: a case study

The epigenetic therapy of cancers is rapidly emerging as an effective approach to chemotherapy as well as to the chemoprevention of cancer and is the focus of intense structure-based drug-design efforts. Histone lysine methylation is one of the pivotal signaling pathways in chromatin-regulatory mechanisms, amongst the array of covalent histone modifications. Lysine histone methyltransferases (HMTases) are transcriptional regulators that target specific lysines on H3 and H4, and can transfer up to three methyl groups on histone tails [27]. Lysine methylation or any of the other histone modifications can have both activating and repressive functions in transcriptional events. An increasing number of epigenetic modifiers have been identified as oncogenes and implicated in the onset of numerous cancers and associated with tumor aggressiveness or prognosis. Reducing or modulating DNA and/or histone epigenetic modifiers activity through specific small molecules appears promising to help suppressing cancer growth. Several high profile targets such NSD2/MMSET has been identified; the structures of most of these targets however remain unknown [28]. Thus, the epigenetic therapy of cancers is still in its infancy due to the lack of available structures for designing specific and selective inhibitors. Epigenetic modifiers enzymes are usually large and complex proteins with several functional domains such as zinc-fingers and PWWP domains that are troublesome during recombinant protein expression and purification for instance. Several crystal structures of the catalytic domain SET alone contributed to shed some light on the histone lysine methylation mechanism. While these structures are of significant interest from a standpoint of drug discovery, they do not provide enough information for an effective drug-design. Particularly, the catalytic SET domain of several HMTase oscillates between an open and a close conformation that are critical for the binding of specific inhibitors. This oscillation mechanism has been described for a few HMTase but a common mechanism has yet to be found amongst the rather large and heterogeneous family of HMTase [28,29]. In addition, the regulation of the putative substrate specificity through the binding of protein partners is not yet understood at the structural level. While crystal structures of valuable drug-targets are being pursued, the use of models in drug discovery is steadily increasing to extend our understanding of histone modifiers.

**Figure 3:**
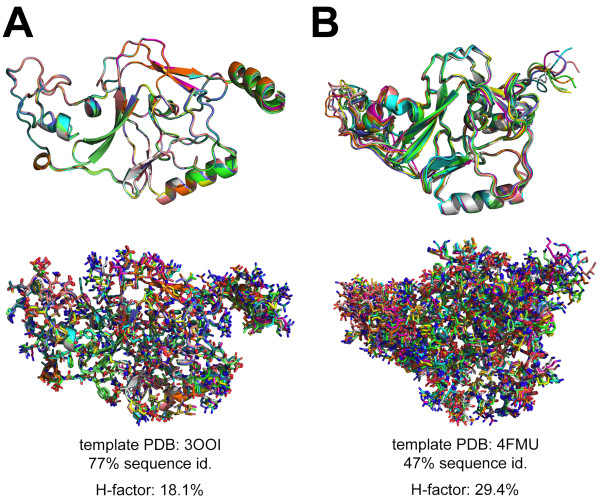
**Case study: modelling of the SET domain of MMSET/NSD2 A.** Structural overlay of a set of 10 models of NSD2-SET modelled using PDB 3OOI (NSD1-SET) as a template. **B**. Structural overlay of a set of 10 models of NSD2-SET modelled using PDB 4FMU (SETD2-SET) as a template. The models are represented as ribbons (top) and with the amino acid side-chains (bottom). The sequence identity between the template and NSD2-SET is indicated along with the H-factor.

Models of epigenetic modifiers utilized in virtual ligand screening efforts have yielded mixed results and only few high-affinity inhibitors have been reported so far. The main obvious issue comes from the errors associated with the models that prevent accurate ligand screening. Errors in homology models can be alleviated with a careful evaluation using the H-factor for instance. In Figure 3, we illustrate the errors in homology modelling by comparing two sets of models for the catalytic domain SET of human histone methyl transferase MMSET. The crystal structure of the SET domain of NSD1 has been recently solved and it is the ideal template to model its close sibling NSD2-SET [30]. Modeling of NSD2-SET based on the NSD1-SET structure is straightforward as both share 77% sequence identity and are biochemically very closely related (Figure 2-A). The structural overlay of ten models build with Modeller v9.11 shows very little backbone deviation highlighting the good match between sequences and structural elements. This represents “a best case scenario” for homology modeling where all the models are highly homologous from the stance of Cα-backbone deviations to the side chains positioning standpoint. Although the crystal structure of NSD2-SET is unknown, the tightly homologous models build with a H-factor of 18.1% tend to indicate a trustworthily set of models for virtual ligand screening. The SET domain is the catalytic region of NSD2 and the low observed deviations of both the backbone and the side-chains increase the likelihood of success in isolating small molecules inhibitors. Figure 3-B describes the modeling of NSD2-SET with the second best template available, the HMTase SETD2. The SET domains of NSD2 and SETD2 share 47% sequence identity together and both have the same biological function at the chromatin (methylation of the same histone mark H3K36) which may justify the use of SETD2-SET (PDB 4FMU) as a modeling template. In addition, the choice of SETD2-SET can possibly be justified as it provides a model for NSD2-SET in a different conformation. Models build with PDB 4FMU as a template have a H-factor of 29.4% and are significantly more heterogeneous than models build with PDB 3OOI as a template. The overall backbone traces differ notably between Figure 3-A and 3-B. It is clear that despite falling into the category of good template (47% sequence identity, same family and same biological function), PDB 4FMU most likely differ substantially from the structure of NSD2-SET and a H-factor of 29.4% captures clearly the differences between the two sets of models (Figure 3-A and 3-B). The set of models for NSD2-SET build with PDB 4FMU cannot be use for accurate analysis such as virtual ligand screening. Drug discovery relies on finding small molecules that should satisfy the empirical Lipinsky rule of 5 in order to maximize bioavailability and drug-delivery. Notably, molecules should have a molecular weight less than 500 Da, low numbers of hydrogen bond donors and acceptors along with a low number of rotatable bonds. To satisfy these stringent requirements while being biologically relevant, the models or structures used in virtual ligand screening should be accurate. A mere Cα-backbone or side chain deviation or >1 Å would dramatically reduce the likelihood of finding biologically active molecules. The models in Figure 3-B illustrate this point. 

## Conclusion

The H-factor does not provide a universal solution to the problem of asserting the quality of a model generated by homology modeling. Our current implementation does not take into account multiple templates, rather only one single framework. The structural components included in the H-factor (i.e. scores (**3**) and (**4**)) are based on the backbone of the models, and do not take into account sidechains and possible errors in their modeling. Second, the scoring function (**3**) of the H-factor measures the heterogeneity of a set of models generated with the same input. It means, that the H-factor cannot be computed on a singular model. In homology modeling the heterogeneity of models can be seen as a quality indicator and building only one single model is not recommended. One of the originalities of the H-factor is the scoring function (**4**). It has been designed to evaluate the biological relevance of the models by comparing the model conformations of all the functional domains in the protein considered with the existing siblings deposited in the Protein Data Bank.

While we acknowledge that there is room for improvement, it remains that the H-factor is a first step in the direction of validating homology models for the biologists in addition to existing methods, as proved in the examples shown above. The four independent scoring functions (**1**, **2**, **3**, **4**) provide the biologists with clear and easy to follow indicators to optimize or correct their models.

## Competing interests

The authors declare that they have no competing interests.

## Authors' contributions

Conceived and designed the experiments: EDL & PK. Performed the experiments: EDL. Analyzed the data: EDL & PK. Wrote the paper: EDL & PK. All authors read and approved the final manuscript.
